# Complete Chloroplast Genome Sequence and Annotation of the Tropical *japonica* Group of Asian Cultivated Rice (*Oryza sativa* L.)

**DOI:** 10.1128/genomeA.01703-15

**Published:** 2016-02-18

**Authors:** Shuo Wang, Li-zhi Gao

**Affiliations:** a Faculty of Environmental Science and Engineering, Kunming University of Science and Technology, Kunming, China; b Faculty of Life Science and Technology, Kunming University of Science and Technology, Kunming, China

## Abstract

We announce here the first complete chloroplast genome sequence of the tropical *japonica* rice, along with its genome structure and functional annotation. The plant was collected from Indonesia and deposited as a germplasm accession of the International Rice GenBank Collection (IRGC 66630) at the International Rice Research Institute (IRRI). This genome provides valuable data for the future utilization of the germplasm of rice.

## GENOME ANNOUNCEMENT

Rice (*Oryza sativa* L.), as an important staple crop of the family *Poaceae*, is distributed widely across diverse tropical-to-temperate regions of both hemispheres and provides the vast majority of daily caloric intake for half the world’s population. It is also well known for its great genetic diversity within species (1, 2), which can be categorized into five distinct varietal groups: *indica*, *aus/boro*, *aromatic* (basmati/sadri), temperate *japonica*, and tropical *japonica* (alias *javanica*) (3–6). Along with the arrival of genome sequencing era, the *indica* and temperate *japonica* rice became some of the first few crop species having their nuclear, chloroplast, and mitochondrial genomes completely sequenced (7–12), which consolidated their valuable status as model systems for other grass species.

The tropical *japonica* rice (*O. sativa* subsp. tropical *japonica*) was most likely domesticated in northern parts of Southeast Asia or South China, and then introduced southward to Southeast Asia and from there to West Africa and Latin American countries (13). Here we first announce its complete chloroplast genome sequence and functional annotations. The plant was collected from Indonesia and deposited as a germplasm accession of the International Rice Collection (IRGC 66630) at the International Rice Research Institute (IRRI), which was then prepared for sequencing as an accession of the 3,000 Rice Genomes Project (3K RGP [14]). Genomic DNA was extracted by a modified cetyltrimethylammonium bromide (CTAB) method at IRRI and shipped to BGI-Shenzhen for the construction of Illumina index libraries. After quality control, at least 3 µg of genomic DNA was randomly fragmented by sonication and size fractionated by electrophoresis. DNA fragments of approximately 500 bp in length were then purified, labeled with 6-bp nucleotide multiplex identifiers, and followed by pooling prior to library construction for next-generation sequencing (NGS). Each library was sequenced in six or more lanes on the Illumina HiSeq 2000 platform to generate 90-bp paired-end reads. The reads were subsequently extracted based on the corresponding unique nucleotide multiplex identifiers and filtered by deleting those with adapter contamination or containing >50% low-quality bases (quality value, ≤5) (14). 

Approximately 1.5 Gb of Illumina paired-end total DNA sequencing data (GigaScience database [15]) were filtered with NCBI-blast version 2.2.31+ (ftp://ftp.ncbi.nih.gov/blast/) to obtain chloroplast DNA reads. The filtered chloroplast DNA reads were then subjected to SOAP*denovo*2 (16), ABySS version 1.9.0 (17), and SPAdes version 3.1.0 (18) for several runs of assembly. The final assembly resulted in a complete circular genome sequence with a length of 134,536 bp and G+C content of 39.7%. Annotation was performed with Dual Organellar GenoMe Annotator (DOGMA [19]) using default parameters to predict protein-coding genes, tRNA genes, and ribosomal RNA (rRNA) genes. For genes with low sequence identity, manual annotation was performed to determine the positions of start and stop codons depending on the translated amino acid sequence using the chloroplast/bacterial genetic code.

### Nucleotide sequence accession numbers.

The source accession for this DNA sample at IRRI is IRGC 66630 (http://iris.irri.org/germplasm2/id/365929), and the accession number for the genetic stock attached to the DNA sample is IRGC 126310 (http://iris.irri.org/germplasm2/id/3059145). The complete chloroplast genome sequence with all genes annotated has been submitted to GenBank under the accession number KT289404.
